# Effectiveness of Efavirenz-Based Regimens in Young HIV-Infected Children Treated for Tuberculosis: A Treatment Option for Resource-Limited Settings

**DOI:** 10.1371/journal.pone.0055111

**Published:** 2013-01-25

**Authors:** Janneke H. van Dijk, Catherine G. Sutcliffe, Francis Hamangaba, Christopher Bositis, Douglas C. Watson, William J. Moss

**Affiliations:** 1 Macha Research Trust, Macha Hospital, Choma, Zambia; 2 Department of Immunology and Infectious Diseases, Erasmus University, Rotterdam, The Netherlands; 3 Department of Epidemiology, Bloomberg School of Public Health, Johns Hopkins University, Baltimore, Maryland, United States of America; 4 Greater Lawrence Family Health Center, Lawrence, Massachusetts, United States of America; 5 Department of Pediatrics, University of Maryland School of Medicine, Baltimore, Maryland, United States of America; University of Cape Town, South Africa

## Abstract

**Background:**

Antiretroviral treatment (ART) options for young children co-infected with HIV and tuberculosis are limited in resource-poor settings due to limited data on the use of efavirenz (EFV). Using available pharmacokinetic data, an EFV dosing schedule was developed for young co-infected children and implemented as the standard of care at Macha Hospital in Southern Province, Zambia. Treatment outcomes inchildren younger than 3 years of age or weighing less than 10 kg receiving either EFV-based ART plus anti-tuberculous treatment or nevirapine-based (NVP) ART were compared.

**Methods:**

Treatment outcomes were measured in a cohort of HIV-infected children seeking care at Macha Hospital in rural Zambia from 2007 to 2010. Informationon the diagnosis and treatment of tuberculosis was abstracted from medical records.

**Results:**

Forty-five children treated for tuberculosis initiated an EFV-based regimen and 69 children initiated a NVP-based regimen, 7 of whom also were treated for tuberculosis. Children receiving both regimens were comparable in age, but children receiving EFV started ART with a lower CD4^+^ T-cell percentage and weight-for-age z-score. Children receiving EFV experienced increases in both CD4^+^ T-cell percentage and weight-for-age z-score during follow-up, such that levels were comparable to children receiving NVP after two years of ART. Cumulative survival after 12 months of ART did not differ between groups (NVP:87%;EFV:80%;p = 0.25). Eleven children experienced virologic failure during follow-up.The adjusted hazard ratio of virologic failure comparing EFV to NVP was 0.25 (95% CI:0.05,1.24) and 0.13 (95% CI:0.03,0.62) using thresholds of 5000 and 400 copies/mL, respectively.Five children receiving EFV were reported to have had convulsions after ART initiation compared to only one child receiving NVP (p = 0.04).

**Conclusions:**

Despite poorer health at ART initiation, children treated for tuberculosis and receiving EFV-based regimens showed significant improvements comparable to children receiving NVP-based regimens. EFV-based regimens should be considered for young HIV-infected children co-infected with tuberculosis in resource-limited settings.

## Introduction

The dual burden of human immunodeficiency virus (HIV) infection and tuberculosis represents a significant threat to the health of children in sub-Saharan Africa. An estimated 3.4 million children worldwide are infected with HIV [1], the majority of whom live in sub-Saharan Africa.In areas of high HIV prevalence, as many as half of incident pediatric tuberculosis cases occur in children infected with HIV [2]. Tuberculosis is among the most common causes of persistent lung disease in HIV-infected children older than 3 years [3], is one of the leading causes of death from respiratory illness in HIV-infected children [4], and accelerates HIV disease progression [5].

Because of the poor prognosis in young children infected with HIV and tuberculosis, there is no alternative to concurrent treatment of both infections [6]. Simultaneous initiation of both therapies increases the risk of immune reconstitution syndrome, but extensive delays in starting antiretroviral therapy (ART) should be avoided. Current WHO recommendations for co-infected children are that ART should be initiated 2–8 weeks after starting treatment for tuberculosis [6], and a cohort study suggested that ART should not be delayed more than 60 days [7].

The optimal antiretroviral regimen for children receiving anti-tuberculous treatment has not been established. Rifampicin is a potent inducer of the cytochrome P450 system and hepatic glucuronidation, resulting in significant reductions in serum levels of several antiretroviral drugs [8]. The alternative, rifabutin, has fewer drug interactions but is often not available in most resource-limited settings, has not undergone formal pharmacokinetic and safety studies in children, and is associated with corneal deposits and other ocular toxicity in children [9]. The preferred antiretroviral regimen for co-administration with rifampicin in adults and older children is two nucleoside reverse transcriptase inhibitors (NRTI) plus efavirenz (EFV). For children younger than 3 years of age receiving rifampicin, current WHO recommendations for antiretroviral therapy include two NRTI plus nevirapine (NVP) or three NRTI [6]. However, both of these options are problematic and have been associated with reduced virologic efficacy compared to other regimens [10], [11], [12], [13].

The product labeling for EFV includes dosages only for children older than 3 years of age and weighing greater than 10 kg, as EFV dosing for younger or smaller children had not been established. The 2006 [14] and 2008 [15] WHO recommendations followed the weight-band dosing table in product labeling (approximately 15 mg/kg/day). However, EFV clearance is not linearly proportional to weight and data are emerging that higher dosages may be required in children older than 3 years of age [16], [17], [18], [19].Children younger than 3 years of age may require even higher relative dosages. In the P1021 trial, which assessed the efficacy of a once-daily regimen containing didanosine, emtricitabine and EFV, serum EFV levels in children younger than 3 years of age were within the therapeutic range when given a fixed dosage of 390 mg (median 47 mg/kg) [20], [21], significantly higher than current recommendations. In the P1070 study, a non-linear weight band dosing scheme averaging approximately 40 mg/kgwas used in African and Asian children younger than 3 years of age. Drug levels in the target range were achieved in the majority of children, except those with the slow-metabolizer CYP2B6516TT genotype who had higher drug levels [22].

Given the limited antiretroviral treatment options in resource-constrained settings for children receiving rifampicin, and the need to initiate ART as soon as possible to avoid excess morbidity and mortality, an EFV dosing schedule extrapolated from available data was developed for the clinical care of young children with tuberculosis. We assessed the effectiveness of EFV-based regimens by comparing treatment outcomes between young co-infected children receiving both anti-tuberculous therapy and EFV-based ART regimens and young children with and without tuberculosis receiving NVP-based ART regimens enrolled in an observational cohort study.

## Methods

### Ethics Statement

The study was approved by the Ministry of Health of the Government of Zambia, the Research Ethics Committee of the University of Zambia and the Institutional Review Board of the Johns Hopkins Bloomberg School of Public Health. Written informed consent was obtained from parents or guardians and assent was obtained from children 8–16 years of age.

### Setting and Clinical Care

The study was conducted at the pediatric HIV clinic at Macha Hospital in rural Southern Province, Zambia. The study setting and population were described in detail elsewhere [23], [24]. Briefly, Macha Hospital is a district-level referral hospital that has provided care to over 7500 HIV-infected adults and children since 2005. HIV care services, including antiretroviral treatment, are provided through the Government of Zambia’s antiretroviral treatment program, with support from the President's Emergency Plan for AIDS Relief (PEPFAR) through the non-governmental organization, AIDSRelief. Care and treatment are provided free of charge by medical doctors and clinical officers. Mothers and infants are provided drugs to prevent mother to child transmission (PMTCT) according to WHO guidelines [25]. Children diagnosed with HIV infectionare determined to be eligible for ART according to the WHO treatment guidelines [6], [14], [15]. Standard ART regimens consist of stavudine or zidovudine plus lamivudine, and a non-nucleoside reverse transcriptase inhibitor (NVP or EFV).

Young children suspected of having tuberculosis undergo a physical examination and chest radiograph. The clinical diagnosis of tuberculosis is based on the results of these examinations and the judgment of the health care provider. Children with tuberculosis are treated with isoniazid (6 months), rifampicin (6 months), and pyrazinamide (2 months). Children treated with rifampicin and eligible for ART are treated with two NRTI and EFV. An EFV dosing schedule based on available data [16], [17], [18], [19], [20], [21], [26]was provided to clinics supported by AIDSRelief throughout Zambia beginning in 2006 and adopted as the standard of care atMacha Hospital for young children with tuberculosis. The schedule included a fixed dosage of 300 mg daily (using scored 600 mg tablets) for children weighing between 4 and 20 kg.

Young children without tuberculosis and eligible for ART were treated with two NRTI and NVP. NVP was dosed using the WHO 2006 dosing recommendations, which included guidance on induction and maintenance dosing [14].

### Study Procedures

Beginning September 2007, HIV-infected children younger than 16 years of age and seeking care at the pediatric HIV clinic at Macha Hospital were eligible for enrollment into an observational cohort study. This report describes a subset of these subjects. Children were evaluated at study visits approximately every three months, at which time a questionnaire was administered to obtain information on demographics, household characteristics and medical history. The child was examined to measure height and weight, and a blood specimen was obtained to measure CD4^+^ T-cell counts and percentages (Guava Easy CD4 system;Guava Technologics, Inc., Hayward, CA) and ALT (ReflotronPlusChemistry Analyzer and CobasC111;Roche Molecular Systems)as part of clinical care. Plasma levels of HIV RNA were quantified by reverse transcriptase polymerase chain reaction assay (Amplicor HIV-1 Monitor v. 1.5, Roche Molecular Systems; lower limit of detection of 400 copies/mL) as part of the study. For children receiving ART, adherence was assessed by pillcounts and syrup volume measurements. For children who missed study visits, home visits were attempted to ascertain their status.

Information regarding prior and current diagnosis and treatment of tuberculosis and adverse events while receiving ART were abstracted from medical records. Adverse events were defined as any clinical sign or symptomor elevated ALT measure possibly or probably related to ART. Elevated ALT measures were graded according to WHO guidelines [6].

### Study Population

**Figure 1:**
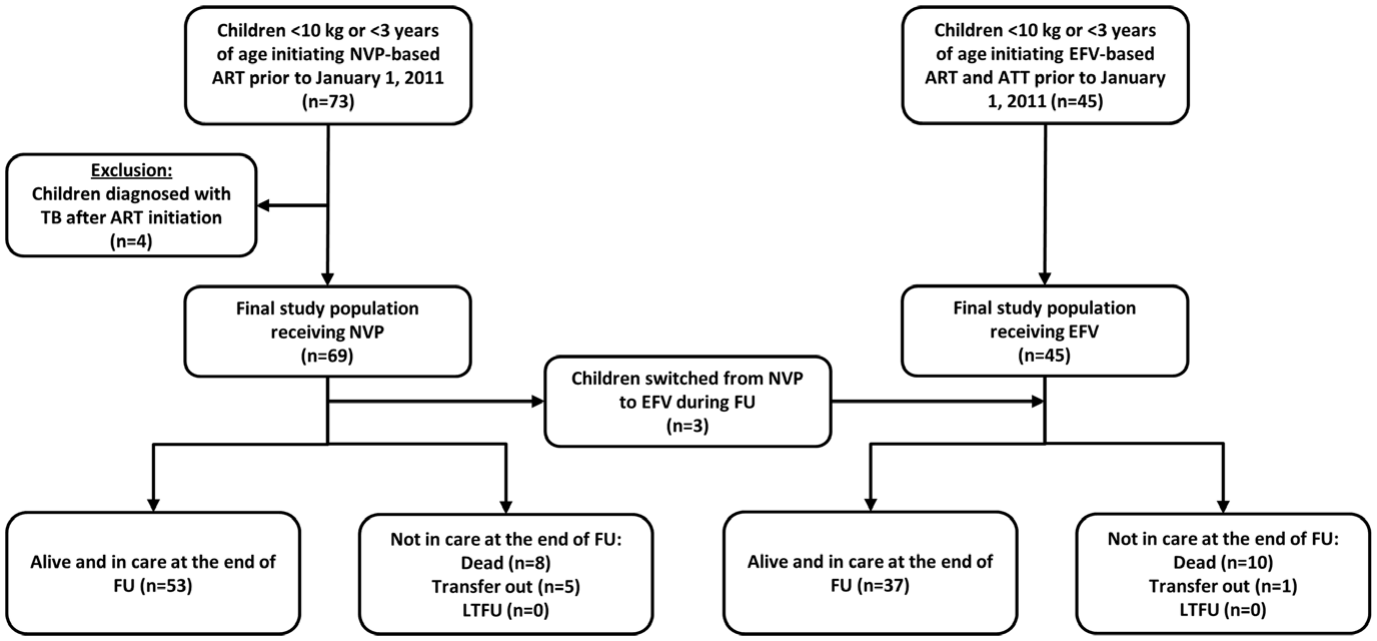
Study flowchart. ART: antiretroviral treatment; ATT: anti-tuberculous treatment; EFV: efavirenz; FU: follow-up; LTFU: loss to follow-up; NVP: nevirapine.

This analysis was restricted to children younger than 3 years of age or weighing less than 10 kg who were enrolled in the observational cohort study and initiated ART with a regimen consisting of two nucleoside analogues plus either EFV or NVP prior to January 1, 2011 (Figure 1). The group of children receiving EFV consisted solely of those receiving concurrent treatment for HIV and tuberculosis. The group of children receiving NVP included children with and without tuberculosis. The children with tuberculosis were inadvertently initiated on a regimen containing NVP and were switched to EFV at the discretion of the clinic physician or clinical officer. Children initiating ART with NVP who were subsequently diagnosed with tuberculosis (n = 4) were excluded. Children were categorized as receiving an EFV or NVP-based regimen according to their regimen at initiation or during follow-up.

Study outcomes, including mortality, virologic failure, CD4^+^ T-cell percentage, growth and adherence were assessed until May 1, 2011. Children were included in the analysis until they died, were lost to follow-up or were administratively censored on May 1, 2011. Children whose last study visit occurred more than six months prior to May 1, 2011 were considered lost to follow-up.

### Statistical Analysis

Data were entered in duplicate using EpiInfo (Centers for Disease Control and Prevention) and analyses were conducted using SAS for Windows version 9.1 (SAS Institute Inc., Cary, NC) and STATA, version 9 (StataCorp LP, College Station, TX). Characteristics of children receiving NVP and EFV at ART initiation were compared using chi-square tests for binary variables and Wilcoxon rank sum tests for continuous variables. Severe immunosuppression was defined by age according to the 2006 WHO treatment guidelines [14]. Weight-for-age (WAZ) z-scores were calculated based on WHO growth standards [27], and children with WAZ below −2 were considered underweight. Children with hemoglobin <8 g/dL were considered severely anemic [28]. Use of drugs by the mother or child to prevent mother to child transmission (PMTCT) was ascertained by interview and confirmed by review of medical records.If measurements were not available from the visit at which ART was initiated, results within 3 months prior to the date of initiation were used.

Clinical and immunologic outcomes were evaluated among children with at least one measurement after ART initiation using longitudinal data analysis. Linear mixed effects models with random intercept, exchangeable correlation structure and robust standard error estimation were used. Interaction terms between EFV and time were included to determine whether trajectories of the outcomes differed between children receiving EFV or NVP. For CD4^+^ T-cell percentage, a spline term was added at 6 months as trajectories were not linear over time.

Survival after ART initiation was evaluated using Kaplan Meier survival curves. Survival curves for children receiving NPV and EFV were compared using the log-rank test. EFV, the primary exposure of interest, was treated as a time-varying covariate as three children who initiated ART with NVP and were receiving anti-tuberculous therapy at ART initiation subsequently switched to EFV.Cox proportional hazards models were used to compare the risk of death between children receiving NVP and EFV.

Virologic outcomes also were evaluated. The proportion of children with viral suppression, defined as a viral load below the limit of detection (400 copies/mL), was calculated for each visit after ART initiation and compared between children receiving NPV and EFV using chi-square tests. As for mortality, virologic failure was evaluated using Kaplan Meier survival curves and Cox proportional hazards models with EFV treated as a time-varying covariate. Virologic failure was defined according to WHO guidelines [6] as at least two viral load measurements >5000 copies/mL among children who received at least six months of ART, and was defined on the date of the second measurement. An alternate definition of virologic failure was also assessed using a cut-off of ≥400 copies/mL. Children entered the analysis on their first viral load measure at or beyond 6 months of ART and were included until they experienced virologic failure, were lost to follow-up or were administratively censored on May 1, 2011.

For all analyses, characteristics known to be associated with the outcome from the published literature or found to be associated with the outcome (p<0.10) in the crude models were considered for inclusion in the multivariable models.

Caregivers were instructed to bring all unused medications at each visit and adherence was measured by pill count or measurement of liquids for each drug prescribed. Adherence measures were capped at 100%. For children taking more than one drug, the adherence percentage of the drug to which the patient was least adherent was used. Children were defined as adherent using two thresholds, depending upon whether they took more than 90% or 95% of drugs prescribed. The proportions of children receiving NVP or EFV who were adherent at each visit and at all visits were compared using chi-square tests.

## Results

### Characteristics of the Study Population

Between September 2007 and December 2010, 114 children younger than 3 years of age or weighing less than 10 kg initiated antiretroviral treatment and were eligible for analysis, including 45 children receiving an EFV-based regimen and 69 children receiving a NVP -based regimen.

Among children receiving EFV, the median time between the start of anti-tuberculous therapy and initiation of ART was 1.9 months (IQR: 1.0, 2.4; range 0.6–5.4). Twenty-eight (62%) children started ART during the intensive phase of anti-tuberculous therapy (within the first two months), and 17 (38%) during the continuation phase (within 2–6 months; median 2.4 months). Among children receiving NVP, five children were previously treated for tuberculosis but started ART after completion of anti-tuberculous therapy. Seven children receiving NVP were also receiving anti-tuberculous therapy at ART initiation (three initiated ART during the intensive phase and four during the continuation phase) and three subsequently switched to EFV (time to switch: 0.4, 0.9, and 1.3 months after ART initiation).

The median age at ART initiation was 17.4 months for children receiving EFV and 20.2 months for children receiving NVP, and the majority of children were female (Table 1). Few children receiving either EFV or NVP had previous exposure to antiretroviral drugs as part of the PMTCT program and the majority of children received an ART backbone of stavudine and lamivudine. Children receiving EFV were significantly more likely to be classified as WHO stage 3 or 4, and have a lower CD4^+^ T-cell percentage, weight and WAZ. They were marginally more likely to have a lower hemoglobin level (Table 1).

**Table 1:** Characteristics of children receiving nevirapine and efavirenz at ART initiation.

	N (NVP/EFV)	Children receiving NVP	Children receiving EFV	p-value
Age in months: median (IQR)	69/45	20.2 (11.0, 27.1)	17.4 (13.6, 22.6)	0.36
Male: n (%)	69/45	31 (44.9)	17 (37.8)	0.45
Mother and/or child received drugs to prevent mother-to-child transmission of HIV (confirmed or self-reported): n (%)	69/45	6 (8.7)	6 (13.3)	0.21
WHO stage 3 or 4: n (%)	32/36	25 (78.1)	36 (100.0)	0.01
CD4%: median (IQR)	64/41	18.5 (15.7, 25.2)	14.2 (9.8, 20.7)	0.007
Severe immunosuppression^a^: n (%)		41 (64.1)	29 (70.7)	0.48
Hemoglobin (g/dL): median (IQR)	66/44	9.4 (8.6, 10.3)	9.0 (7.9, 9.8)	0.08
Weight (kg): median (IQR)	69/45	8.8 (7.2, 10.0)	7.2 (6.2, 8.6)	0.005
Weight-for-age z-score: median (IQR)	69/45	−1.7 (−2.8, −0.5)	−2.7 (−3.6, −1.8)	0.001
Underweight^b^: n (%)		32 (46.4)	31 (68.9)	0.02
BCG vaccination scar present: n (%)	69/45	65 (94.2)	41 (91.1)	0.53
Regimen: n (%)	69/45			
Stavudine/lamivudine		59 (85.5)	33 (73.3)	
Zidovudine/lamivudine		9 (13.0)	10 (22.2)	
Abacavir/lamivudine		1 (1.5)	2 (4.4)	0.25

BCG: Bacillus Calmette-Guerin; EFV: efavirenz; IQR: interquartile range; NVP: nevirapine; WHO: World Health Organization.

a Severe immunosuppression defined by age according to the 2006 WHO treatment guidelines.

b Underweight defined as weight-for-age z-score less than −2.

At the end of follow-up, 8 (12.1%) children receiving NVP and 10 (20.8%) receiving EFV died (p = 0.21), and 5 (7.6%) children receiving NVP and one (2.1%) receiving EFV transferred to another clinic (p = 0.19). No child was lost to follow-up. The median duration of follow-up on ART was comparable between groups, with 13.4 months (IQR: 5.9, 27.0) of follow-up for children receiving NVP and 16.7 months (IQR: 8.2, 23.3) for children receiving EFV (p = 0.68).

### Clinical and Immunologic Outcomes

**Figure 2:**
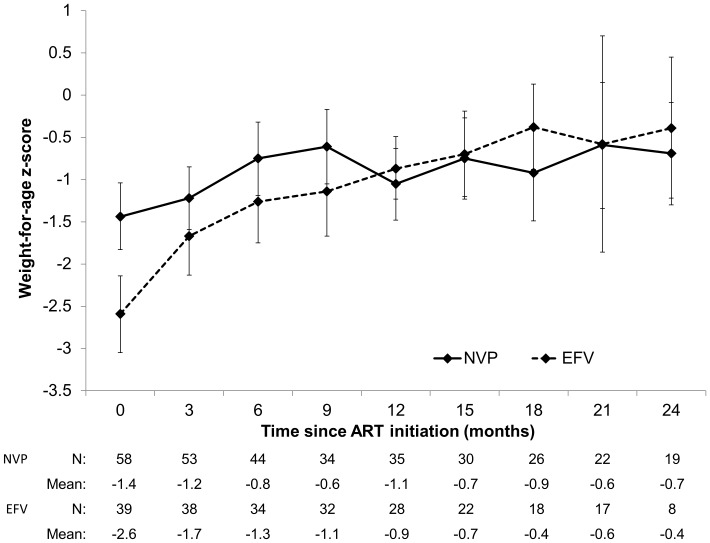
Mean weight-for-age z-score (95% confidence interval) after ART initiation by regimen.

Children receiving EFV initiated ART with a significantly lower WAZ than children receiving NVP, and experienced significantly greater increases in WAZ during follow-up (NVP: mean change +0.1, standard deviation [SD] 1.0; EFV: +1.8, SD 1.6, at 12 months; p<0.0001) (Figure 2). Results of the longitudinal data analysis showed significantly different trajectories of WAZ between the two groups of children, with children receiving EFV experiencing a significantly greater increase in WAZ per month, such that they were able to catch-up to children receiving NVPwithin two years of ART (Table 2).

**Table 2:** Changes in CD4^+^ T-cell percentages and weight-for-age z-scores after ART initiation by regimen.

	Crude			Adjusted		
	Children receiving NVP	Children receiving EFV	p-value	Children receiving NVP	Children receiving EFV	p-value
***CD4^+^ T-cell percentage (CD4%)***			a			b
CD4% at ART initiation (SE)	22.61 (1.09)	18.42 (1.08)	0.006	25.41 (1.93)	22.79 (2.01)	0.12
Increase in CD4% per month in first 6 months of ART (SE)	2.04 (0.17)	1.49 (0.21)	0.05	2.04 (0.18)	1.51 (0.21)	0.06
Increase in CD4% per month after 6 months of ART (SE)	−0.11 (0.10)	0.37 (0.10)	0.001	−0.11 (0.10)	0.36 (0.11)	0.001
***Weight-for-age z-score (WAZ)***			a			c
WAZ at ART initiation (SE)	−1.25 (0.17)	−2.04 (0.21)	0.005	−1.07 (0.25)	−1.41 (0.25)	0.009
Increase in WAZ per month (SE)	0.02 (0.01)	0.07 (0.02)	0.003	0.02 (0.01)	0.07 (0.02)	0.008

EFV: efavirenz; NVP: nevirapine; SE: standard error.

a Results shown are from linear mixed effects models with random intercept, exchangeable correlation structure and robust standard error estimation. Interaction terms between EFV and time were included to determine whether trajectories of the outcomes differed between children receiving EFV or NVP. For CD4^+^ T-cell percentage, a spline term was added at 6 months as trajectories were not linear over time.

b Adjusted for hemoglobin, weight-for-age z-score, and age at ART initiation.

c Adjusted for hemoglobin, CD4^+^ T-cell percentage, weight-for-age z-score, and age at ART initiation.

**Figure 3:**
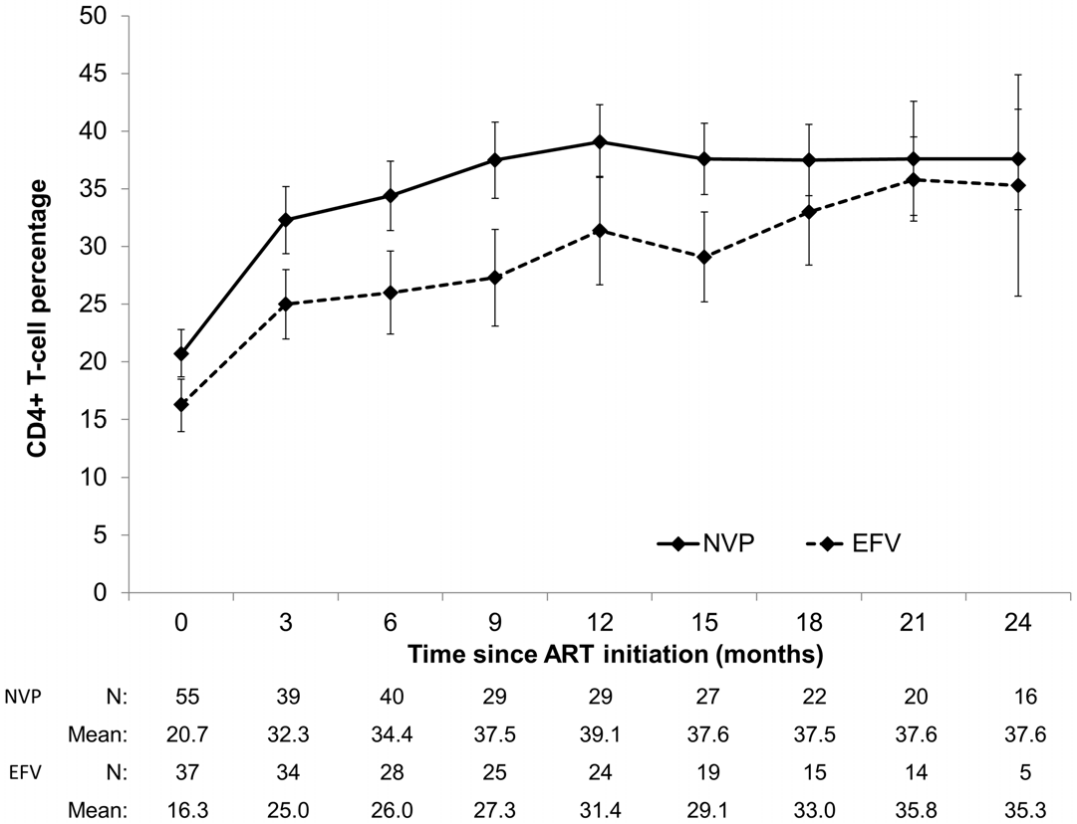
Mean CD4^+^ T-cell percentage (95% confidence interval) after ART initiation by regimen.

Children receiving EFV also initiated ART with a significantly lower CD4^+^ T-cell percentage than children receiving NVP, but experienced comparable increases in CD4^+^ T-cell percentage during follow-up (NVP: +16.9%, SD 8.4; EFV: +15.0%, SD 9.6 at 12 months; p = 0.47) (Figure 3).Results of the longitudinal data analyses showed significantly different trajectories of CD4^+^ T-cell percentages between the two groups of children. Among children receiving NVP, CD4^+^ T-cell percentage increased rapidly in the first 6 months of ART and then stabilized for the duration of follow-up (Table 2). In contrast, among children receiving EFV, CD4^+^ T-cell percentage increased more slowly in the first 6 months but continued to increase for the duration of follow-up, such that levels were comparable among all children after two years of ART (Table 2).

### Virologic Failure

**Figure 4:**
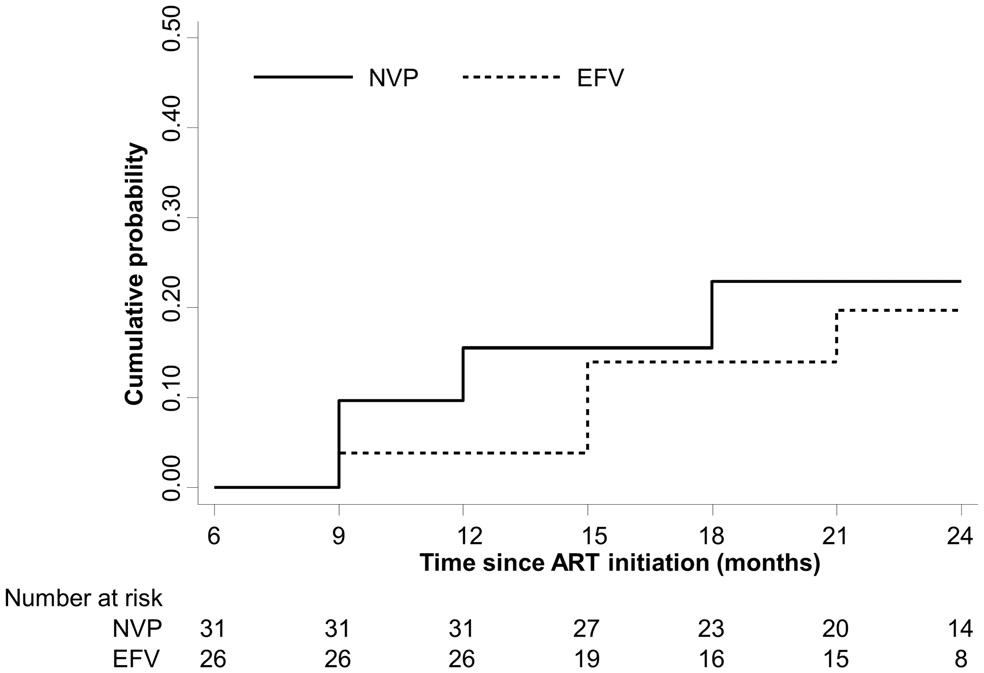
Cumulative probability of virologic failure after 6 months of ART by regimen.

Within the first 3 months of ART, the majority of children receiving NVP (80.7%) and EFV (87.5%; p = 0.50) achieved virologic suppression. The majority of children maintained virologic suppression at 12 (NVP: 78.8%; EFV: 91.7%; p = 0.19) and 24 months (NVP: 68.4%; EFV: 77.8%; p = 0.61) of ART. Virologic failure was assessed among the 72 children (40 receiving NVP and 32 receiving EFV) with at least two viral load measures at or beyond 6 months of ART. Four children receiving EFV (12.5%) and 7 children receiving NVP (17.5%; p = 0.56) experienced virologic failure (Figure 4; log-rank test: p = 0.63; Table S1). None of the children receiving NVP who experienced virologic failure were also receiving anti-tuberculous therapy at ART initiation.The risk of virologic failure was not significantly different among children receiving EFV compared to children receiving NVP (hazard ratio [HR]: 0.73; 95% CI: 0.21, 2.49). After adjusting for CD4^+^ T-cell percentage and WAZ at ART initiation, receipt of PMTCT, and number of viral load measures, the risk of virologic failure was lower among children receiving EFV (adjusted HR: 0.25; 95% CI: 0.05, 1.24; Table 3), although this result was not statistically significant. When virologic failure was defined by two viral load measures above the lower limit of detection (400 copies/mL) after 6 months of ART, the percentage of children with virologic failure was 15.6% among children receiving EFV compared to 22.5% among children receiving NVP (adjusted HR: 0.13; 95% CI: 0.03, 0.62; Table 3).

**Table 3:** Crude and Adjusted models for virologic failure.

	Virologic failure threshold of 5000 copies/mL		Virologic failure threshold of 400 copies/mL	
	Crude HR (95% CI)	Adjusted a HR (95% CI)	Crude HR (95% CI)	Adjusted a HR (95% CI)
EFV	0.73 (0.21, 2.49)	0.25 (0.05, 1.24)	0.72 (0.24, 2.15)	0.13 (0.03, 0.62)
CD4% at ART initiation (per 5)	0.83 (0.54, 1.28)	0.60 (0.34, 1.06)	0.76 (0.51, 1.13)	0.53 (0.31, 0.91)
WAZ at ART initiation				
≥ −2	1	1	1	1
−2.1 to −3	0.56 (0.11, 2.81)	0.56 (0.11, 2.93)	0.40 (0.08, 2.00)	0.37 (0.07, 1.95)
< −3	1.47 (0.37, 5.88)	4.21 (0.69, 25.69)	1.81 (0.55, 5.92)	7.48 (1.36, 41.01)
Receipt of PMTCT	2.05 (0.44, 9.50)	8.37 (0.90, 78.30)	1.28 (0.28, 5.91)	9.61 (1.03, 89.39)

ART: antiretroviral therapy; EFV: efavirenz; HR: hazard ratio; PMTCT: prevention of mother-to-child transmission; WAZ: weight-for-age z-score.

a Additionally adjusted for number of viral load measures.

### Mortality

**Figure 5:**
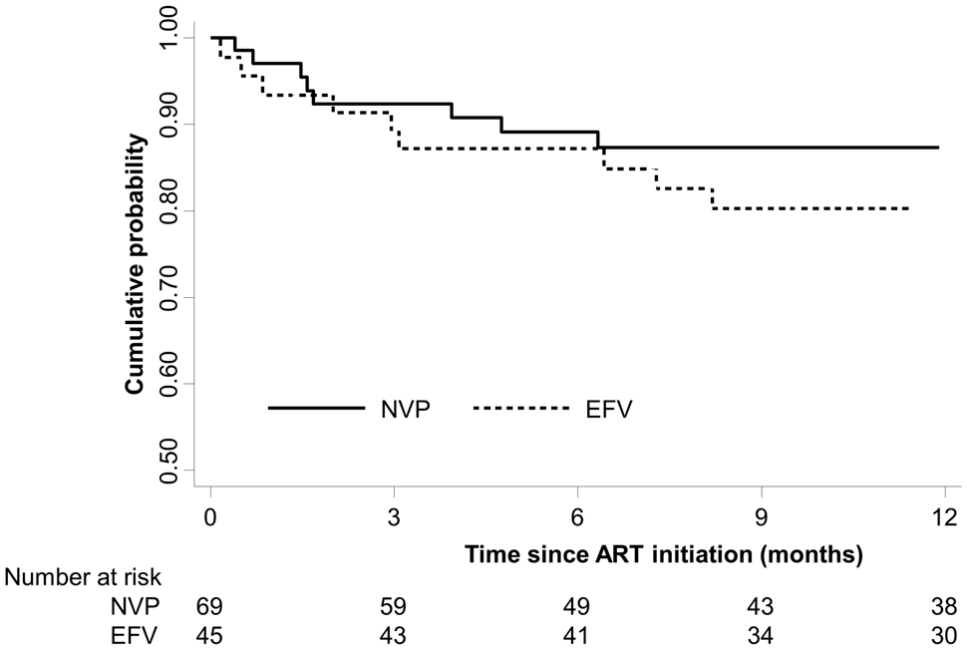
Cumulative survival after ART initiation by regimen.

Eighteen deaths were recorded among study children (NVP: 12.1%; EFV: 20.8%; p = 0.21). Among children who died, the median time to death after ART initiation was 1.6 months (IQR: 1.1, 4.3) among children receiving NVP and 3.4 months (IQR: 0.9, 7.3) among children receiving EFV (p = 0.41). Cumulative survival was high at 6 months (NVP: 89%, 95% CI: 79, 95; EFV: 87%, 95% CI: 74, 94) and 12 months (NVP: 87%, 95% CI = 76, 93; EFV: 80%, CI = 65, 89) after initiating ART and did not differ significantly between groups (Figure 5; log-rank test: p = 0.25). The mortality rate per 100 person-years was 8.71 (95% CI: 4.36, 17.41) among children receiving NVP and 15.81 (95% CI: 8.51, 29.38) among children receiving EFV. The risk of mortality was non-significantly higher among children receiving EFV (HR: 1.72; 95% CI: 0.68, 4.36). After adjusting for CD4^+^ T-cell percentage, WAZ and hemoglobin at ART initiation, no difference in mortality was observed (adjusted HR: 1.00; 95% CI: 0.30, 3.31).

### Adherence

The percentage of children who took more than 90% of their dispensed medication at all visits, verified by pill count and syrup measurement, was 56% for children receiving NVP and 46% for children receiving EFV (p = 0.35). No significant differences were observed in adherence between children receiving NVP or EFV at any of the study visits (Table S2). Similar results were obtained when adherence was defined as taking more than 95% of dispensed medication.

### Adverse events Associated with Antiretroviral Therapy

None of the children discontinued EFV or NVP because of adverse events. Five children receiving EFV were reported to have had convulsions after ART initiation compared to only one child receiving NVP (p = 0.04). Convulsions were reported 2 weeks to 9 months after ART initiation. No children were prescribed antiepileptic drugs. The dose of EFV was reduced in one child whose caregiver reported intermittent seizures at one clinic visit 3 months after ART initiation.The child was reported to have had no more convulsions, was switched to NVP after completing anti-tuberculous treatment, and died at home five months later with symptoms of gastroenteritis and pneumonia. In the other five children, including one child with convulsions reported before and after EFV initiation, three children whose convulsions were suspected to be related to febrile episodes (2 receiving EFV, 1 receiving NVP), and one child with suspected HIV encephalopathy, treatment was continued without modification and the seizures did not recur during the period of observation. No other differences in clinical symptoms were found between children receiving EFV and NVP. Twenty-two children had transiently elevated alanine aminotransferase levels (ALT ≥62.5 U/L [6]) during follow-up: 29.4% of children receiving NVP and 19.4% of children receiving EFV (p = 0.29). The median time from ART initiation to the first episode of elevated ALT was 30 weeks (IQR: 26, 49; range 2–129) among children receiving NVP and 49 weeks (IQR: 28, 76; range: 23–76) among the children receiving EFV. All episodes were grade 1 or 2 events and no child required a change or discontinuation of treatment as a result of the elevated ALT.

## Discussion

Despite poorer health at ART initiation, children younger than 3 years of age who were treated for tuberculosis and received an EFV-based ART regimen showed significant improvements in clinical, immunological and virologic outcomes, comparable to young children receiving a NVP-based regimen.

To our knowledge, no studies of the virologic efficacy of EFV-based regimens have been conducted among children younger than 3 years of age to support recommendations for its use in this population. However, use of EFV-based regimens for young children co-infected with tuberculosis would be a useful alternative treatment strategy given the limited treatment options available.WHOcurrently recommends that HIV-infected infants and children younger than 3 years of age and treated for tuberculosis receive either two NRTI plus NVP or three NRTI [6]. However, there are significant drawbacks to both options. When co-administered with rifampicin, studies have found NVP levels to be significantly reduced in both adults [29], [30], [31] and children [32], [33], thereby increasing the likelihood of drug resistance and virologic failure. Increasing the dose of NVP when co-administered with rifampicin may achieve target drug levels [31], [34], but may also lead to unacceptable toxicity and discontinuation rates [14], [34].There is also increasing evidence that NVP is inferior to EFV and other regimens in terms of virologic efficacy among adults and children, with [35], [36] and without [11], [12], [13], [37]tuberculosis. Regimens comprising 3 NRTI are also problematic, as they have been associated with high rates of virologic failure in both children [10] and adults [38], particularly when baseline viral loads exceed 100,000 copies RNA/mL [39], [40]. In co-infected children, who are likely to have high baseline viral loads, the risk of such failure is likely to be unacceptably high.

An alternative treatment option not endorsed by the WHO for young children with tuberculosis is a regimen consisting of 2 NRTI plus ritonavir-boosted lopinavir (LPV/r). As with NVP, lopinavir levels are significantly reduced by rifampicin [8]. Doubling the dose of LPV/r to overcome this pharmacokinetic interaction has resulted in high toxicity and discontinuation rates [41], or persistently inadequate serum concentrations [42].One small study in South African children found that increasing the dose of ritonavir to achieve a LPV/r ratio of 1∶1 resulted in acceptable pharmacokinetics for most children with little reported toxicity [43]. However, ritonavir as a single agent is not yet widely available in many resourced-limited settings and is associated with poor tolerability [44].

Consequently, there is need for alternative treatment strategies for young children with tuberculosis and data to support their use. The dosing schedule for EFV in Zambia was developed based on available pharmacokinetic data [16], [17], [18], [19], [20], [21], [26] and was independent of the observational cohort study. Children with tuberculosis receiving EFV-based regimens in this study achieved good clinical and immunologicoutcomes that were comparable to children receiving NVP-based regimens, most of whom were not co-infected with tuberculosis. Similar to studies in adults [35], [36], our findings suggest that children receiving EFV-based regimens were more likely to achieve virologic suppression compared to children receiving NVP-based regimens.Children with tuberculosis receiving EFV-based regimens had higher mortality compared to children receiving NVP-based regimens, although the difference was not statistically significant within the limited power conferred by the few number of deaths. This difference was presumably due to the poorer clinical and immunologic state of the children with tuberculosis, and was not observed after adjusting for these factors. In other studies, co-infection with tuberculosis was associated with increased mortality in children receiving ART [45].

All children tolerated EFV and, in contrast to other studies [20], [46], [47], [48], [49], no child discontinued use during the period of observation. Due to the young age of the study population, symptoms were assessed by caregiver report and many symptoms possibly related to EFV use, including loss of concentration, sleep disorders, or psychotic reactions, were difficult to evaluate. Caretakers and guardians were asked about symptoms and complaints in routine clinical care but not specifically about possible adverse events related to ART or EFV, which may have resulted in underreporting of side effects. However, if adverse events did occur and were missed by the guardian and healthcare worker, they were likely mild and transient. ALT was the only laboratory measure assessed during follow-up; however, the relevance of elevated ALT measurements is unclear as they occurred without symptoms and only sporadically in most children.

The reports by parents or caretakers of a seizure in five of the children receiving EFV and one receiving NVP are concerning but a causal association is difficult to establish in this observational study. In preclinical studies of EFV, convulsions were seen in monkeys with high EFV levels [50]. Only a single case of seizures related to EFV use in children has been reported [51] in a child who developed absence seizures in association with high levels of EFV and a slow-metabolizer genotype. All children with seizures reported here continued their drug (one with EFV dose reduction) without further report of seizures, and given the limited diagnostics available, the contribution of ART to the seizures is difficult to determine. The greater frequency of the CYP2B6 TT genotype associated with slow metabolism in Africans [52], and the median half-life of only 11.4 hours in young children that makes relatively large dosages necessary to achieve adequate trough levels, means that some children will have transient high drug levels. Shortening the dosing interval to 12 hours in small children is a potential strategy to avoid high peak levels that might lead to toxicity.

Although informative and encouraging, this study has several limitations. This was an observational cohort study and the diagnosis of tuberculosis and decisions regarding ART regimens were made by the treating clinicians. These decisions were independent of the observational cohort study from which data for this report were abstracted, and there was no provision for pharmacokinetic studies or comprehensive safety monitoring. With the implementation of the EFV dosing schedule at the HIV clinic, children with tuberculosis were prescribed an EFV-based ART regimen. Consequently, the characteristics of the children receiving EFV-and NVP-based ART regimens were different, as the majority of children receiving NVP-based regimens were not co-infected with tuberculosis. Attempts were made to account for these differences in the analysis but measures of all potentially relevant characteristics were not available. The diagnosis of tuberculosis in children is difficult and radiographic or microbiologic tests were not performed on all children in the study. We attempted to address this issue by excluding children diagnosed with tuberculosis after ART initiation but could not account for children with undiagnosed tuberculosis during the study period. Additional limitations include the small sample size, which limited the power to detect statistically significant differences between the two groups (particularly for virologic outcomes), the relatively short duration of follow-up, and, as previously described, the difficulties in measuring EFV-related side effects.

### Conclusions

This is the first study to demonstrate that EFV can be used effectively in young HIV-infected children with tuberculosis. Additional studies will be required to validate and optimize an EFV dosing strategy for young children co-infected with tuberculosis. Given the increasing number of young HIV-infected children starting ART in sub-Saharan Africa, the high burden of tuberculosis, the limited treatment options in this region, and the limited virologic efficacy of NVP, use of EFV in young children should be considered.

## Supporting Information

Table S1 **Characteristics of children with virologic failure.**
(DOCX)

Table S2 **Adherence to antiretroviral therapy by month and regimen.**
(DOCX)
