# Supplementary material for: Effectiveness of Efavirenz-Based Regimens in Young HIV-Infected Children Treated for Tuberculosis: A Treatment Option for Resource-Limited Settings
Source: PLoS One. 2013 Jan 25;8(1):e55111. doi: 10.1371/journal.pone.0055111 (PMC3555823; doi:10.1371/journal.pone.0055111)
Supplement: Table S1 — Characteristics of children with virologic failure. (DOCX) [file pone.0055111.s001.docx]

**Table S1. Characteristics of children with virologic failure**

|  | **Characteristics at ART initiation** | | | | | | | | **Characteristics at virologic failure^a^** | | |
| --- | --- | --- | --- | --- | --- | --- | --- | --- | --- | --- | --- |
|  | **Age (mths)** | **Sex** | **PMTCT** | **ART Regimen** | **CD4%** | **WAZ** | **Viral load (copies/mL)** | **TB diagnosed** | **Duration of ART (mths)** | **Age (mths)** | **CD4%** |
| 1 | 21.4 | F | No | D4T/3TC/NVP | 15.6 | -0.34 | 2000 | No | 8.7 | 30.1 | 31.3 |
| 2 | 29.5 | F | No | AZT/3TC/NVP | 18.9 | -2.35 | 216000 | Yes: ART started after ATT | 16.8 | 46.3 | 22.6 |
| 3 | 28.7 | M | No | D4T/3TC/NVP | 17.5 | -2.29 | 214000 | No | 17.9 | 46.7 | 42.9 |
| 4 | 20.2 | F | No | D4T/3TC/NVP | 14.0 | 0.23 | --- | No | 9.4 | 29.6 | 43.8 |
| 5 | 22.9 | F | No | D4T/3TC/NVP | 19.3 | -1.22 | 758000 | Yes: ART started after ATT | 8.8 | 31.6 | 39.6 |
| 6 | 7.8 | F | Yes^b^ | D4T/3TC/NVP | 28.2 | -0.88 | 255000 | No | 12.3 | 20.1 | 33.0 |
| 7 | 4.2 | M | Yes^c^ | D4T/3TC/NVP | 21.8 | -0.53 | 750000 | No | 11.2 | 15.4 | 20.9 |
| 8 | 9.5 | M | No | ABC/3TC/EFV | 18.0 | -5.47 | --- | Yes: ART started during intensive phase | 15.8 | 25.4 | 12.7 |
| 9 | 13.6 | F | No | D4T/3TC/EFV | 18.8 | -3.03 | 750000 | Yes: ART started during continuation phase | 10.1 | 23.7 | 30.2 |
| 10 | 10.1 | F | No | D4T/3TC/EFV | 9.2 | -1.75 | 750000 | Yes: ART started during intensive phase | 20.9 | 31.0 | 31.0 |
| 11 | 33.6 | F | No | D4T/3TC/EFV | 6.9 | -3.99 | 750000 | Yes: ART started during intensive phase | 14.8 | 48.5 | 15.0 |

3TC: lamivudine; ABC: abacavir; ART: antiretroviral therapy; ATT: anti-tuberculous treatment; D4T: stavudine; EFV: efavirenz; FTC: emtricitabine; NVP: nevirapine; PMTCT: prevention of mother-to-child transmission; TB: tuberculosis; TEN: tenofovir; WAZ: weight-for-age z-score

^a^ Virologic failure defined according to WHO guidelines as at least two viral load measurements >5000 copies/mL among children receiving at least 6 months of treatment. Failure was defined at the second measurement.

^b^ Mother received shortcourse HAART (AZT/3TC/NVP) for 2 months; Baby received single dose NVP and AZT for 7 days

^c^ Mother received HAART (FTC/TEN/NVP) starting in the third trimester; Baby received NVP and AZT for 7 days
