# Supplementary material for: Effectiveness of Efavirenz-Based Regimens in Young HIV-Infected Children Treated for Tuberculosis: A Treatment Option for Resource-Limited Settings
Source: PLoS One. 2013 Jan 25;8(1):e55111. doi: 10.1371/journal.pone.0055111 (PMC3555823; doi:10.1371/journal.pone.0055111)
Supplement: Table S2 — Adherence to antiretroviral therapy by month and regimen. (DOCX) [file pone.0055111.s002.docx]

**Table S2. Adherence to antiretroviral therapy by month and regimen**

|  | **% with Adherence >95%** | | | | | **% with Adherence >90%** | | | | |
| --- | --- | --- | --- | --- | --- | --- | --- | --- | --- | --- |
| **Month after ART initiation** | **N** | **Children receiving NVP** | **N** | **Children receiving EFV** | **p-value** | **N** | **Children receiving NVP** | **N** | **Children receiving EFV** | **p-value** |
| 3 | 50 | 66.0 | 36 | 61.1 | 0.64 | 50 | 76.0 | 36 | 75.0 | 0.92 |
| 6 | 42 | 78.6 | 31 | 58.1 | 0.06 | 42 | 81.0 | 31 | 74.2 | 0.49 |
| 9 | 35 | 74.3 | 29 | 69.0 | 0.64 | 35 | 85.7 | 29 | 79.3 | 0.50 |
| 12 | 32 | 81.3 | 25 | 68.0 | 0.25 | 32 | 87.5 | 25 | 76.0 | 0.26 |
| 15 | 27 | 74.1 | 19 | 57.9 | 0.25 | 27 | 81.5 | 19 | 63.2 | 0.16 |
| 18 | 25 | 88.0 | 17 | 64.7 | 0.07 | 25 | 96.0 | 17 | 94.1 | 0.78 |
| 21 | 22 | 81.8 | 15 | 86.7 | 0.69 | 22 | 86.4 | 15 | 93.3 | 0.50 |
| 24 | 19 | 89.5 | 9 | 55.6 | 0.04 | 19 | 94.7 | 9 | 88.9 | 0.57 |

ART: antiretroviral therapy; EFV: efavirenz; NVP: nevirapine
